# Feral Pig Populations Are Structured at Fine Spatial Scales in Tropical Queensland, Australia

**DOI:** 10.1371/journal.pone.0091657

**Published:** 2014-03-10

**Authors:** Jobina Lopez, David Hurwood, Bart Dryden, Susan Fuller

**Affiliations:** 1 Science and Engineering Faculty, Queensland University of Technology, Brisbane, Queensland, Australia; 2 Terrain Natural Resource Management Limited, Innisfail, Queensland, Australia; Université de Sherbrooke, Canada

## Abstract

Feral pigs occur throughout tropical far north Queensland, Australia and are a significant threat to biodiversity and World Heritage values, agriculture and are a vector of infectious diseases. One of the constraints on long-lasting, local eradication of feral pigs is the process of reinvasion into recently controlled areas. This study examined the population genetic structure of feral pigs in far north Queensland to identify the extent of movement and the scale at which demographically independent management units exist. Genetic analysis of 328 feral pigs from the Innisfail to Tully region of tropical Queensland was undertaken. Seven microsatellite loci were screened and Bayesian clustering methods used to infer population clusters. Sequence variation at the mitochondrial DNA control region was examined to identify pig breed. Significant population structure was identified in the study area at a scale of 25 to 35 km, corresponding to three demographically independent management units (MUs). Distinct natural or anthropogenic barriers were not found, but environmental features such as topography and land use appear to influence patterns of gene flow. Despite the strong, overall pattern of structure, some feral pigs clearly exhibited ancestry from a MU outside of that from which they were sampled indicating isolated long distance dispersal or translocation events. Furthermore, our results suggest that gene flow is restricted among pigs of domestic Asian and European origin and non-random mating influences management unit boundaries. We conclude that the three MUs identified in this study should be considered as operational units for feral pig control in far north Queensland. Within a MU, coordinated and simultaneous control is required across farms, rainforest areas and National Park Estates to prevent recolonisation from adjacent localities.

## Introduction

Feral pigs (*Sus scrofa*) are one of the most widespread pest animals in Australia causing significant agricultural, environmental and economic damage. They prey on newborn lambs, and trample and consume crops [1]–[4], and cost the agricultural industry alone more than $100 million per annum [5]. Feral pigs also spread endemic diseases and are potentially a threat to domestic livestock and human health as they are vectors of a range of serious exotic diseases [6]–[10]. Environmental damage by feral pigs is primarily through modification of native habitat, but predation and competition with native animals has also been reported [11]–[14].

### Feral pigs in tropical north Queensland

Feral pigs pose a significant threat to World Heritage biodiversity values and agriculture including sugarcane and tropical fruit crops in far north Queensland. It is estimated that two to three million feral pigs inhabit north Queensland, with a density of approximately three pigs per km^2^ in the Wet Tropics World Heritage Area (WHA) [15]. Anecdotal reports suggest that feral pigs routinely move between WHA rainforest and agricultural crops, particularly during the dry season. However, radio-tracking of feral pigs in the WHA has revealed that pigs are generally sedentary and move only short distances (approximately one km on average) from the centre of their home ranges [16]. The same study reported an average home range size of eight km^2^, but seasonal differences were observed with dry season home ranges more than twice the size of wet season home ranges. Furthermore, Mitchell et al. [16] reported that feral pigs on the rainforest boundary permanently reside in the rainforest/crop eco-tone, have home range sizes that expand into the agricultural crop zones according to seasonal influences (dry season) and it is this population that seems to support the community perception of feral pig migration in the dry season to the lowlands to forage on crops and then returning to the highlands in the wet season. Recent research using stable isotope analysis of hair samples supports this finding as pigs captured in sugarcane or on the crop-rainforest interface were found to use both habitats and switched regularly between forest and crop resources [17]. Pigs in remote rainforest however, appear to meet their dietary needs solely from rainforest resources. Currently there is no data to suggest that large scale migration is a common phenomenon in tropical regions [16], [18].

### Identification of management units

To develop an efficient control program to reduce or eradicate a pest population, demographic connectivity among locations needs to be understood [19] in order to identify effective management units (MUs). MUs have been defined based on significant allelic frequency differentiation (i.e. significant *F*_ST_) at neutral nuclear and/or mitochondrial DNA (mtDNA) loci [20], [21] among sample locations. Operational units for feral pig control have been designed traditionally for practical operational purposes (according to geography, government jurisdiction and landholder agreement), however poor design may result in only a short term reduction in numbers as pigs reinvade from uncontrolled areas of the broader population [22]. Biological knowledge of natural populations allows the identification of demographically independent units with clusters of subpopulations that need to be eradicated simultaneously in order to maximize the long-term success of the operation [23]. When there is no *a priori* knowledge of population boundaries, molecular data analysed in a landscape ecology framework [21] can be used to delineate the spatial scale of demographic independence and to infer population units for management.

Molecular studies on the ecology of feral pigs in south-west Western Australia have identified distinct population structure across river catchments [24], [25] and have provided evidence of illegal translocation [26]. Cowled et al. [22] examined the population structure of a highly controlled pig population in south-west Queensland and found a single population spanning 4000 km^2^. Aerial survey results suggested that there was no reduction in population size after two years of control and indicated that pigs reinvaded the study area and rapidly restored the population to pre-existing levels. In a later study by Cowled et al. [27], a 500000 km^2^ rangeland area was examined and results indicate that rivers and floodplains act as major migration routes for feral pigs and that control over large areas (thousands of kilometers) is required.

### Project Aims

The population structure of feral pigs in tropical regions is currently unknown. This study identified feral pig management units in the Tully-Innisfail region of far north Queensland. In particular, this study determined whether the region acts as a single panmictic, demographically independent management unit or is composed of multiple management units based on the pattern and route of feral pig movement. Only when ecologically meaningful, demographically independent populations have been identified can targeted control programs be implemented on a realistic spatial scale. A long-lasting reduction in feral pig densities is required to protect the biodiversity values of the Wet Tropics WHA of far north Queensland.

## Methods

### Ethics Statement

Trained trap operators provided all the samples used in the project from feral pigs that had been euthanized (shot) as part of a commercial trapping program operating under approved Australian Standard Operating Procedures for feral pig trapping (NSW DPI 2005 http://www.dpi.nsw.gov.au/agriculture/pests-weeds/vertebrate-pests/codes-of-practice/operating-procedures/humane-pest-animal-control). No pigs were trapped and euthanized for the purposes of this study. Feral pigs were trapped by commercial trappers (Boar Busters Ltd) contracted to TerrainNRM who provided samples to this project. Written permission has been obtained from all landholders where feral pig trapping took place and Bart Dryden from Terrain NRM can be contacted for details regarding landholder permission statements. GPS coordinates for sample locations can be found in Table S1. This study was approved by the Queensland University of Technology Animal Ethics Committee (Tissue Notification Approval # 1000000226) and did not involve the use of any endangered or protected species of animal. No trapping occurred in a protected area or National Park.

### Sample Collection

**Figure 1:**
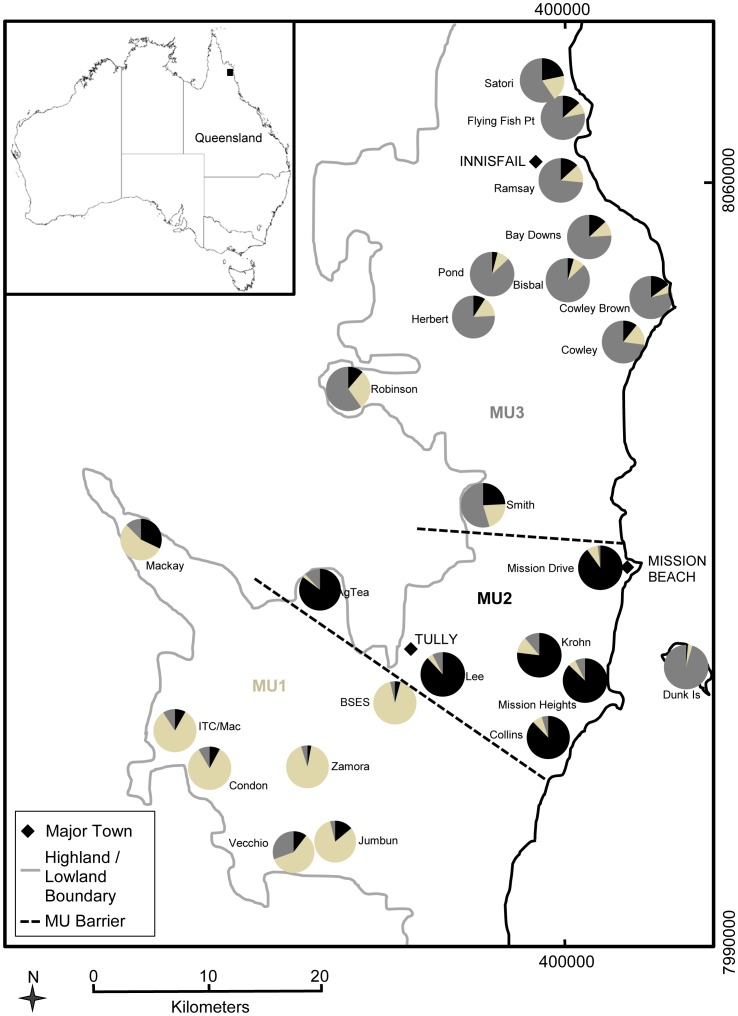
Proportion of individuals at each site with ancestry to each of the three inferred groups (beige, grey and black) based on Bayesian clustering analysis and positioned on a map of the study area.

Feral pig tail and ear tissue samples (N = 328) were obtained from 28 locations (Table S1) between Tully and Innisfail in far north Queensland, Australia (Fig. 1). Demographic data including gender and weight were recorded. The samples were stored in 100% ethanol at -20°C until DNA extraction.

### DNA Extraction

Total DNA from tail and ear tissue samples was extracted using a modified salting out methodology [28]. Each tissue sample was placed in a 1.5 ml tube and digested at 55°C overnight in an incubation buffer (50 mM Tris, 20 mM EDTA and 5% SDS) and 10 µl 20 mg/ml Proteinase K. After complete digestion, the samples were centrifuged for 10 seconds at 13000 rpm and then chilled on ice for 10 minutes. 250 µl of saturated NaCl was added to each sample and inverted several times and chilled for a further 5 minutes on ice. The sample tubes were then centrifuged at 13000 rpm for 15 minutes. Approximately 500 µl of the clear supernatant was collected and placed into a newly labelled tube and 1 ml of AR grade ethanol was added to precipitate the DNA. The samples were placed in a −20°C freezer overnight and then centrifuged at 15000 rpm for 15 minutes. All the supernatant was removed and the DNA pellet was rinsed in 500 µl of cold 70% ethanol and then centrifuged at 15000 rpm for 5 minutes. The supernatant was removed and DNA pellets were left to dry overnight at room temperature and then the DNA was re-suspended in 50 µl of TE buffer and stored at −20°C. A dilution (1∶50) was made and stored at 4°C until used in the PCR.

### Microsatellite Screening

Seven microsatellite loci (S0002, SW240, SW632, SW857, SW911, SW936, SW951) that have been shown to be polymorphic and unlinked in *Sus scrofa* [29] were used in the study. PCR reactions were carried out in a 12.5 µl volume with a PCR reaction mixture of 50–100 ng of template DNA, 0.2 µM of each forward primer, 0.2 µM of each reverse primer with a fluorescent label (FAM, PET, NED, VIC) (Applied Biosystems, USA) at the 5′ end, 1× MyTaq Red buffer, 0.25 unit of MyTaq HS Polymerase and 8.25 µl of ultra-distilled water. PCR conditions included an initial denaturation (94°C) for 5 minutes, and then 30 cycles of 94°C for 15 seconds, annealing temperature (see Hampton *et al.* [24]) for 15 seconds, 72°C for 30 seconds, and a final extension step at 72°C for 4 minutes. A Mastercycler ep (Eppendorf, Hamburg, Germany) was used. For fragment analysis a 1∶5 dilution of amplified PCR products in distilled water was made. From the diluted PCR, 2 µl were added to a master mix solution of 9 µl Hi-Di and 1 µl of 600LIZ size standard. The PCR products from multiple amplification reactions were combined in a single lane (pseudo-multiplexing). Fragment analysis of the DNA was resolved using the ABI3500 genetic analyser (Applied Biosystems, Australia). DNA fragments were scored manually using Genemapper v4.1 (Applied Biosystems, Australia).

### DNA Sequencing

A hyper-variable portion of the mtDNA control region from 34 individuals was amplified using primers MT16498H [30] and MT15996L [31]. The PCR reaction was performed in a 25 µl volume and contained 0.6 µM of each primer, 1× MyTaq Red Buffer (Bioline, Australia), 0.5 unit of My Taq HS DNA polymerase (Bioline, Australia), 16.4 µl of ultra-distilled H_2_O and 50–100 ng of template DNA. A Mastercycler ep (Eppendorf, Hamburg, Germany) was used and the PCR cycle protocol included an initial denaturation (94°C) for 15 minutes, and then 30 cycles of 94°C for 15 seconds, 50°C for 15 seconds, 72°C for 30 seconds, and a final extension at 72°C for 5 minutes. PCR products were purified using an Isolate PCR and Gel Kit (Bioline, Australia) according to manufacturer's instructions. Purified PCR products were amplified in a sequence reaction containing 1.0 µl of PCR product, 1.0 µl of MT15996L (3.2 µM), 0.5 µl of version 3.1 ABI Prism ® Big Dye Terminators (Applied Biosystems, CA, USA), 3 µl of 5× sequencing buffer and 14.5 µl of dH_2_O. The sequencing cycle protocol involved an initial denaturation at 94°C for 5 minutes, followed by 29 cycles of 94°C for 10 seconds, 50°C for 5 seconds, 60°C for 4 minutes, and then a final hold step of 4°C for 10 minutes. Sequenced DNA was precipitated using a standard ethanol/EDTA protocol prior to analysis on an ABI3500 genetic analyser (Applied Biosystems, Australia).

### Data Analysis

Microchecker v2.2.3 [32] (http://www.microchecker.hull.ac.uk/) was used to check for scoring errors, specifically large allele drop-out, stutter bands or presence of null alleles. Exact p values for Hardy Weinberg and linkage disequilibrium at the intra-population level were calculated using a Markov chain method with 1000 dememorisation steps, 100 batches and 1000 iterations per batch for each locus using Genepop on the Web v4.0.10 [33] (http://genepop.curtin.edu.au/). The significance level for each test (Hardy Weinberg p<0.0003; linkage disequilibrium p<0.0001) was adjusted for multiple comparisons using the Bonferroni correction method [34].

Microsatellite allele frequencies and F-statistics were calculated using Arlequin v3.5.1.3 software [35] (http://cmpg.unibe.ch/software/arlequin35/). Probabilities of significant population structure were computed using 100 iterations of a non-parametric permutation process as implemented in Arlequin. Only sites with a minimum sample size of five were included in these analyses. A Bayesian clustering approach implemented in Structure v2.3 [36] (http://pritch.bsd.uchicago.edu) was used to estimate the number of genetic clusters (*K*) in a sample and to assign individuals to one or more of these populations (*k*). Five runs of *K* = 1 to 25 was performed at 100000 MCMC repetitions and 20000 burn-in period using no prior location information, independent allele frequencies and a model of admixture. The posterior probability was then calculated for each value of *K* using the estimated log-likelihood to choose the optimal number of populations using the Evanno *et al.* [37] method implemented in Structure Harvester [38] (http://taylor0.biology.ucla.edu/structureHarvester/). Individuals were assigned to each of the inferred populations based upon the highest percentage of membership (based on the percentage of ancestry that can be attributed to each inferred population).

### Spatial autocorrelation analysis in GenAlEx v6.41 [39]

(http://www.anu.edu.au/BoZo/GenAlEx/) was used to explore the patterns of individual genotypes in space across the entire study site. Populations with less than five individuals were excluded from the analysis. Geographic distances were calculated from Northing and Easting UTM coordinates (Table S1). An autocorrelation was generated that provides a measure of the genetic similarity between pairs of individuals whose geographic separation falls within a specified distance class. The distance class size at which the autocorrelation coefficient is no longer significant provides an approximation of the extent of detectable positive spatial genetic structure.

Thirty-four pigs were selected for mtDNA sequencing and were chosen 1) to provide a broad representation of samples across the study area; 2) because they were individuals assigned in the Structure analysis as a non-resident to the population in which they were sampled; and/or 3) they presented a signature of recent mixed ancestry or poor assignment based on results of the Structure analysis. The mtDNA sequence data was aligned by eye using BioEdit v7.0.0 [40]. The program MEGA v5.1 [41] (http://www.megasoftware.net/) was used to construct a neighbour joining (NJ) tree, using Tamura-Nei distance and 1000 bootstrap replications. Twenty-eight published sequences from Asian domestic, European domestic, Asian wild boar and European wild boar [42]–[44] were obtained from GenBank (Table S2) and were incorporated in the analysis to clarify pig breed origins in the NJ tree.

## Results

### Genetic Differentiation (Microsatellite DNA)

Seven microsatellite loci were screened for 328 feral pigs sampled from 28 sites in far north Queensland. The microsatellite data were tested for null alleles and only one locus (SW857) at one site contained significant homozygote excess. No evidence was found for scoring error stutter bands or large allele dropout. Following Bonferroni correction, no significant deviation from Hardy Weinberg equilibrium was detected. Only three of the 441 pairwise comparisons for deviation from linkage equilibrium were significant following Bonferroni correction, therefore all loci were included in all of the subsequent analyses. The number of alleles detected ranged from four (SW951) to 14 (S0002), while observed heterozygosity ranged from 0.484 (SW632) to 0.814 (SW240).

An indication of the degree of gene flow among feral pigs from different sample sites can be obtained by estimating the level of genetic differentiation among sites using the *F*_ST_ statistic. The highest significant pairwise *F*_ST_ value was 0.28 and was recorded between AgTea and Pond (Table S3). The lowest pairwise *F*_ST_ value was 0 between ITC/Mac and Condon, 8 km apart and between Vecchio and Ramsey, approximately 80 km apart. There was no obvious relationship between genetic and geographic distance i.e. no isolation by distance (Table S3).

**Figure 2:**
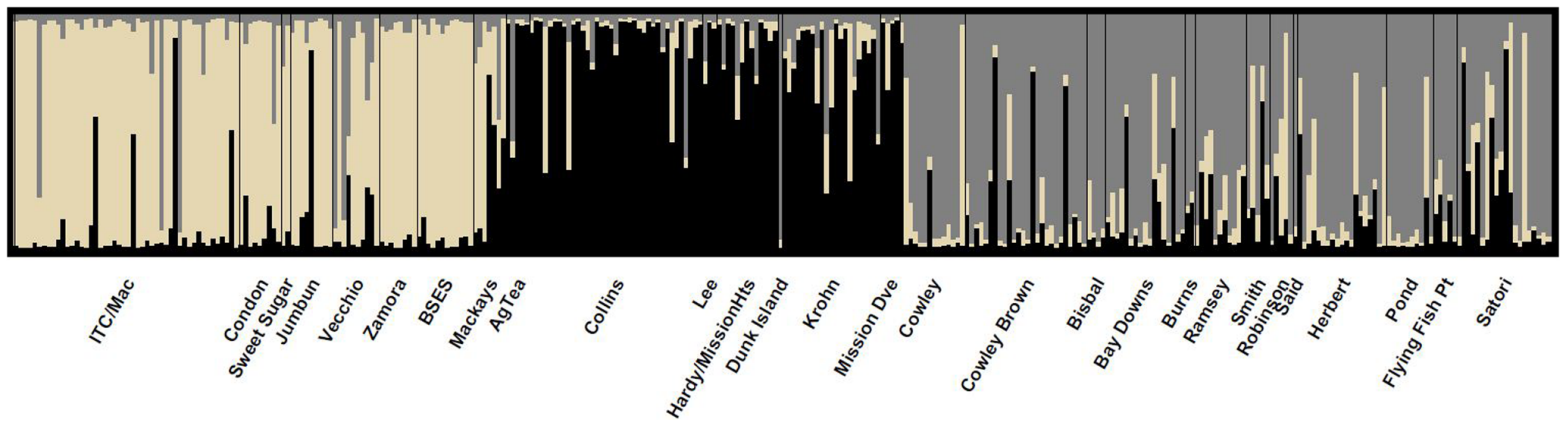
Structure bar plot with each bar representing the proportion of ancestry of each sampled individual to the three inferred groups (beige, grey and black). Labels on the x axis refer to sample sites.

The Structure analysis based on the microsatellite data clearly indicated the presence of population structure, with three groups inferred using the Evanno method. These three groups, represented by the three colours in Fig. 2, indicate that three populations or demographically independent units exist in the study area. MU1 is located to the south of the study area, MU2 centrally and MU3 to the north (Fig. 1). Some very distinct differences exist between sites in neighbouring MUs that do not appear to be related to geographic distance. For example, BSES and Lee are separated by less than five km but exhibit very strong microsatellite differentiation.

**Figure 3:**
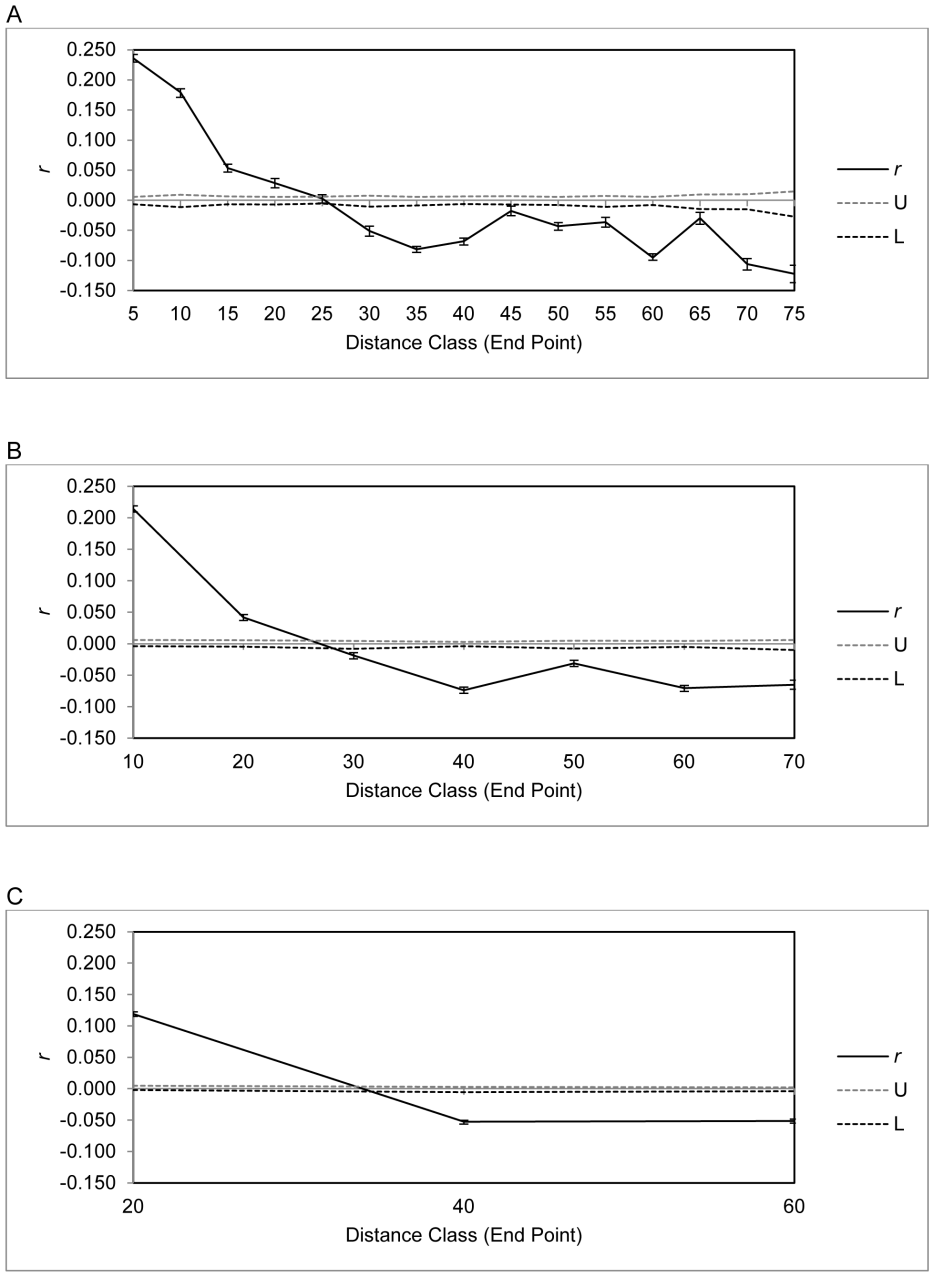
Spatial correlograms showing the autocorrelation coefficient (*r*) as a function of distance, 95% error bars and 95% confidence intervals surrounding the null hypothesis of a random distribution of feral pigs. (A) 5 km Distance Class. (B) 10 km Distance Class. (C) 20 km Distance Class.

Results of the spatial autocorrelation analyses for 5, 10 and 20 km distance classes are displayed in Fig. 3. In Fig. 3A, a 5 km distance class was used, resulting in positive and significant correlation *r* values up to 20 km, and an x intercept of 25.3 km. With a larger distance class of 10 km (Fig. 3B), *r* is still significant and positive at 20 km, with an x intercept of 26.9 km. As the maximum distance between any two sites was 68 km, the maximum distance class that was testable and provided three points on a correlogram was 20 km. At this scale (Fig. 3C), *r* remains significant and positive up to 20 km, and provided an x intercept of 33.9 km. These results indicate that the scale of population structure of feral pigs in the study area to be between approximately 25 and 35 km. No evidence of multiple scales of population structure was found as the correlograms did not show oscillation back and forth across the autocorrelation confidence limits.

The majority of individuals in each MU exhibited pure ancestry (considered >80% assignment to a single cluster; Pritchard *et al.* [36]) to the MU from which they were sampled (Fig. 2). However, a small number of individuals (nine of the 328) clearly exhibited >80% ancestry from a population outside of that from which they were sampled, indicating recent migration or translocation.

### Mitochondrial DNA Analysis

**Figure 4:**
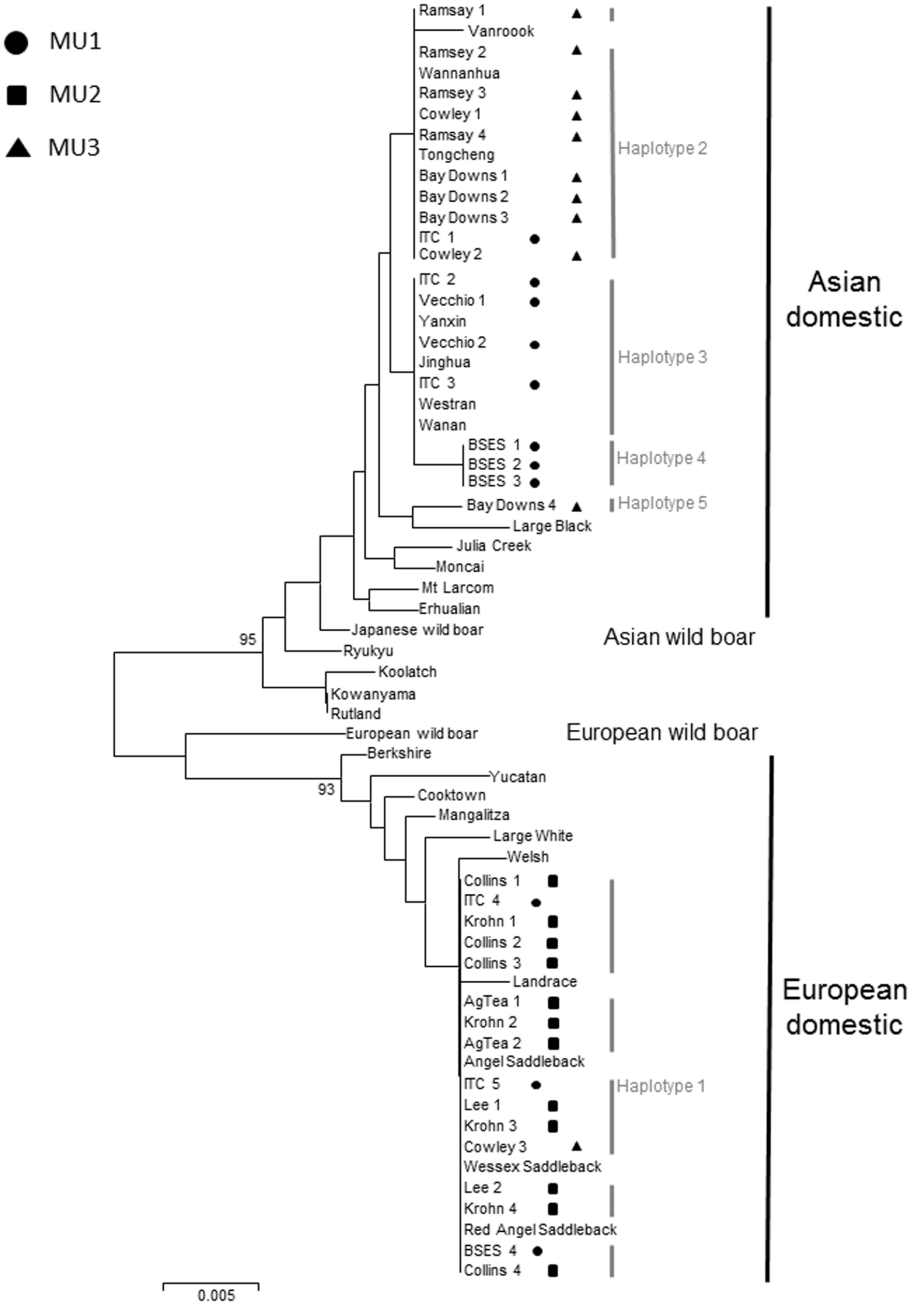
Neighbour joining tree showing the genetic relationships among individuals based on Tamura-Nei genetic distances using control region mtDNA sequence data. Only bootstrap values >90% are shown and the scale indicates genetic distance. Symbols represent management unit designation based on microsatellite Bayesian clustering analysis (• MU1, ▪ MU2, ▴ MU3).

A 405 bp fragment of mitochondrial DNA was sequenced from 34 feral pigs revealing 16 variable sites with a 7∶1 Ti/Tv ratio resulting in the detection of five unique haplotypes (GenBank accession numbers KJ409773 – KJ409777). When aligned with selected homologous sequences available from GenBank (Table S2), all five haplotypes were assigned to one of two well-supported clades that represent Asian and European domestic breeds (see Fig. 4). Only a single haplotype (Haplotype 1) was identified as belonging to the European clade represented by 16 individuals. Among the remaining 18 individuals, four haplotypes (Haplotypes 2–5) were identified as being of Asian domestic origin. Although Asian wild boar ancestry has been detected in feral pigs from northern Australia [45], we found no evidence in this study.

Fig. 4 shows that Haplotype 1 is identical to those identified as Saddleback breeds. Of the Asian haplotypes, Haplotype 2 is identical to Wannanhua and Toncheng breeds, Haplotype 3 is identical to Yanxin, Jinghua, Westran and Wanan breeds and Haplotype 4 is imbedded in this clade (64% bootstrap support). Uncertainty however, surrounds specific breed of origin for Haplotype 5, although they are undoubtedly imbedded in the broader Asian domestic clade.

The neighbour joining cluster analysis based on mitochondrial DNA sequence data from 62 domestic breeds and feral pigs clearly revealed two well supported lineages (95% bootstrap support) corresponding to pigs of Asian domestic origin and European domestic origin (Fig. 4). While MU1 and MU3 were predominately composed of feral pigs of Asian domestic origin (only approximately 20% had European domestic breed ancestry), MU2 contained exclusively pigs originating from European domestic breeds.

Within the Asian domestic lineage sampled in this study there is further division (albeit with poor bootstrap support; 43% and 26% bootstrap support respectively) for the existence of two sub-clades. The first sub-clade consists entirely of Haplotype 2 individuals and is almost exclusively found in MU3 to the north, with the single exception of one individual with this haplotype sampled at ITC from MU1. The second sub-clade (Haplotypes 3 and 4) is solely made up of MU1 individuals sampled to the south. The single individual carrying Haplotype 5 (Bay Downs 4) appears as a sister lineage to these two groupings. While this apparent geographical separation appears consistent with the distribution of MUs identified in the Structure analysis, some anomalies are apparent. For example, ITC 2, Vecchio 1 and Vecchio 2 were sampled in MU1, possessed mtDNA haplotypes consistent with others from MU1 yet had high assignment probability to MU3 (92%, 91% and 87%, respectively).

Of greater significance is the fact that several individuals with European mtDNA were confidently assigned using microsatellite analysis to either MU1 or MU3, where the majority of pigs have Asian mtDNA. Specifically, ITC 4, ITC 5 and BSES 4 sampled from MU1, were assigned to MU1 based on microsatellite data (96%, 77% and 95%, respectively), yet clearly carried the European Haplotype 1 (Fig. 4). Similarly, Cowley 3 was sampled in MU3, was assigned to MU3 with 97% confidence based on microsatellite analysis, yet had European mtDNA. Of the sequenced individuals, none assigned to MU2 had Asian mtDNA.

## Discussion

### Assessing the geographic scale of feral pig population structure

The overall objective of this study was to examine the population genetic structure of feral pigs in far north Queensland. Our results indicate that the study area from south of Tully to north of Innisfail does not constitute a single demographic population, but rather is composed of three discrete management units.

Our results indicate that feral pig MUs in tropical north Queensland exist at a scale of 25 to 35 km. This result is consistent with studies undertaken in Western Australia that described feral pig management units at a scale of 25 km and limited migration between populations [24]. Our results also support the findings of traditional radio-tracking studies in tropical habitats that have found feral pigs to be relatively sedentary, with defined home ranges [16], [18], a result that is consistent with the theory that an animal's home range size will be small in resource abundant habitats. Mitchell *et al.* [16] reported that feral pigs in the Wet Tropics WHA have an average home range size of 8 km^2^ and move an average distance of 1 km. Furthermore, evidence of frequent large scale migration-like movement was not found.

We found evidence that some dispersal or translocation has occurred between MUs. A small number of the sampled feral pigs clearly exhibited ancestry from a MU outside of that from which they were sampled. While feral pigs in the Wet Tropics are generally sedentary [16], Mitchell [15] found that adult boars would sometimes temporarily relocate 10–20 km away from their home range. However, no evidence was found in the current study for male biased dispersal. Instead, putative migrants consisted of juvenile and adult males and females.

Of particular interest are the results for a number of the sites located adjacent to large areas of rainforest (Mackays, Robinson and Smith) that reveal samples with a high proportion of mixed ancestry. This pattern may be because these samples have been founded by an unknown source, providing evidence that the adjacent rainforest may harbour unsampled populations. However, without samples from these areas, this hypothesis cannot be substantiated. Alternatively, individuals at these sites have mixed ancestry as a result of mating between individuals from different MUs and as such, indicate that a higher level of gene flow/movement occurs in these areas (indicated by non-significant *F*_ST_s), potentially along the rainforest-crop boundary. This result concurs with Mitchell *et al.* [16] who found a high degree of pig movement along the rainforest-crop boundary. Feral pigs inhabiting the rainforest-crop boundary had home ranges that were twice as large during the dry season compared with the wet season, as boundary pigs moved further to access resources.

It is important to consider the boundaries of the three MUs, and what natural or artificial barriers may be restricting movement. We cannot confirm the northern boundary of MU3 as the northern extent of sampling in this study coincided with the northern boundary in the Innisfail region, and therefore MU3 may in fact be larger in size. Likewise for MU1; we cannot confirm the southern boundary as it coincided with the most southerly sites that were sampled. However, some interesting results were found for the internal putative boundaries between MUs. For example in MU1, BSES is located north of the Tully River and Zamora and Jumbun are located south of the Tully River, but there is little genetic differentiation between these sites and therefore the Tully River is not a barrier to feral pig movement. In contrast, BSES is genetically distinct from sites located a short distance away on the eastern side of a major highway (eg. Lee, Collins and Krohn). However, sites located either side of the highway in the north in MU3 were not genetically differentiated and it is therefore unlikely that the highway *per se* is a barrier to movement. Potentially, geography (particularly elevation) and land-use may play a role in facilitating or restricting movement and consequently influences MU boundaries. For example in the Tully area, the highway passes close to steep mountains which may limit feral pig movement (resulting in the genetic delineation between BSES and Lee), whereas in the north (MU3) the predominant land-use is agriculture and there are broad flat areas either side of the highway that may facilitate feral pig movement (resulting in no genetic differentiation either side of the highway in MU3). However, another possible but not necessarily mutually exclusive explanation comes from the examination of domestic pig origin revealed in the mitochondrial DNA analysis.

### Origins of feral pigs in Australia

Historical records indicate that the first settlers introduced European domestic pigs to Australia in 1788 [46] and no evidence exists to suggest that *S. scrofa* inhabited Australia prior to the settlement of the Europeans, despite populations being present in the neighbouring Pacific islands. At least three introductions of Asian domestic pigs into Australia have been documented [46]–[48] and European domestic pigs have been crossed with Asian domestic breeds in Europe [49], [50], making it impossible to discern between direct importation of Asian pigs into Australia or introgression via Europe.

Recent molecular studies have corroborated the historical records that pigs of European and Asian domestic ancestry were introduced to Australia. Gongora *et al.* [45] found feral pigs in north-west Queensland with Asian domestic ancestry, European domestic ancestry in pigs from Cooktown in far north Queensland and Asian Ryukyu wild boar ancestry in feral pigs from Cape York Peninsula. However, the origins of feral pigs from the Wet Tropics region south of Cooktown have not been investigated.

Our results suggest that gene flow is restricted among pigs of domestic Asian and European origin and non-random mating influences management unit boundaries. MU2 had feral pigs of European domestic origin, while MU1 and MU3 consist mainly of pigs with Asian domestic origin. So in the case of the two neighbouring sites BSES and Lee located in two different MUs, a physical barrier may not be restricting gene flow, but rather pigs of European domestic origin at Lee are not randomly mating with pigs of Asian domestic origin at BSES. This has not previously been reported in population genetic studies of pigs. Luetkemeier *et al.* [51] found a distinct separation of Asian (wild and domestic) from European (wild and domestic) pigs based on both mitochondrial and microsatellite DNA, however the majority of samples came from captive farm stock. Gongora *et al.* [45] recognised that feral pigs with Asian domestic mitochondrial DNA more commonly displayed one particular allele at the GPIP nuclear locus, while pigs with European domestic mitochondrial DNA displayed the alternative GPIP allele. Heterozygous individuals were also found indicating hybrids are possible. However, this study did not examine pig populations, but rather a single individual from each site and the relative frequency of hybridisation could not be ascertained. Our results suggest that preferential mating among individuals of the same domestic origin occurs frequently, however we do not discount that hybridisation also occurs. Mitochondrial DNA data presented here also indicates that where hybridisation does occur, it is non-random. We see a signature of mtDNA introgression from European into Asian populations but have no evidence for mtDNA gene flow in the other direction. As mtDNA is solely inherited maternally, these data suggest that European females recognise Asian boars as potential mates more readily than Asian females recognise boars of European origin. Significantly more mtDNA sequence data would be required to verify this hypothesis.

While the precise mechanism remains unknown, in farm studies pheromone communication can play an important role in influencing reproductive processes in mammals. In particular, signalling and priming pheromones released by boars affect female receptivity and may be attractants or inducers of sexual activity [52]. Furthermore, feral pigs exhibit considerable morphological variation in body size, shape and coat colour [8], [14], [46], and morphological differences in domestic breeds have been recognized since domestication of the species. Morphological and/or pheromone differences offer a potential explanation for the results found in our study and together with ecological factors (availability of resources and home range size) and geographical and land use variation, a combination of mechanisms is likely to have resulted in the population structure found in this study.

Regarding the introduction history of the populations identified in this study, we can speculate that each MU is likely to represent three separate introductions. According to the mtDNA data, each of the two Asian MUs are almost reciprocally monophyletic and it is unlikely that there has been sufficient time for the respective MUs to have reached this level of differentiation given the relatively short time that pigs have been in north Queensland. The European population was probably established simultaneously, preventing the two Asian populations from readily exchanging genes.

### Management Implications

This study has identified three management units in the Innisfail-Tully region and these MUs should be considered as operational units for feral pig control. Localised control of feral pigs at the property/farm level within an ecological management unit is not likely to be effective in the long term because there is movement among sites within a MU, and recolonisation of controlled areas will occur rapidly. Coordinated feral pig control of all properties within a MU at the same time is required. Results of this study and previous radio tracking research [16] suggest that feral pig control within adjacent rainforest areas and National Park Estates should also be undertaken at the same time, particularly along the rainforest-crop boundary.

## Supporting Information

Table S1 Feral pig sample collection details
(DOCX)

Table S2 Genbank accession numbers and pig breed for control region sequences used in Figure 4
(DOCX)

Table S3 Pairwise *F*_ST_ values. Significant values following Bonferroni correction are highlighted in bold (p<0.0002).
(DOCX)
