# Supplementary material for: Feral Pig Populations Are Structured at Fine Spatial Scales in Tropical Queensland, Australia
Source: PLoS One. 2014 Mar 10;9(3):e91657. doi: 10.1371/journal.pone.0091657 (PMC3948871; doi:10.1371/journal.pone.0091657)
Supplement: Table S1 — Feral pig sample collection details (DOCX) [file pone.0091657.s001.docx]

| **Site** | **Sample Size** | **UTM Northing** | **UTM Easting** |
| --- | --- | --- | --- |
| ITC/Mac | 48 | \| 8011501 \| 363978 \| \| --- \| --- \| | |
| Condon | 9 | \| 8004579 \| 368116 \| \| --- \| --- \| | |
| Sweet Sugar | 2 | \| 8002725 \| 383483 \| \| --- \| --- \| | |
| Jumbun | 9 | \| 8001427 \| 380580 \| \| --- \| --- \| | |
| Vecchio | 10 | \| 7999218 \| 378382 \| \| --- \| --- \| | |
| Zamora | 8 | \| 8006734 \| 377674 \| \| --- \| --- \| | |
| AgTea | 5 | \| 8024047 \| 377751 \| \| --- \| --- \| | |
| BSES | 12 | \| 8011829 \| 387054 \| \| --- \| --- \| | |
| Mackays | 7 | \| 8026867 \| 365440 \| \| --- \| --- \| | |
| Collins | 37 | \| 8008911 \| 396974 \| \| --- \| --- \| | |
| Lee | 3 | \| 8015937 \| 388032 \| \| --- \| --- \| | |
| Hardy/MissionHts | 13 | \| 8016351 \| 403436 \| \| --- \| --- \| | |
| Dunk Island | 1 | \| 11984851 \| 411753 \| \| --- \| --- \| | |
| Krohn | 21 | \| 8015758 \| 399039 \| \| --- \| --- \| | |
| Mission Dve | 4 | \| 8016794 \| 403731 \| \| --- \| --- \| | |
| Cowley | 14 | \| 8043318 \| 404592 \| \| --- \| --- \| | |
| Cowley Brown | 26 | \| 8050804 \| 408260 \| \| --- \| --- \| | |
| Bisbal | 4 | \| 8055896 \| 399565 \| \| --- \| --- \| | |
| Bay Downs | 17 | \| 8070295 \| 399537 \| \| --- \| --- \| | |
| Burns | 2 | \| 8063503 \| 400171 \| \| --- \| --- \| | |
| Ramsey | 11 | \| 8059789 \| 398575 \| \| --- \| --- \| | |
| Smith | 5 | \| 8029761 \| 391710 \| \| --- \| --- \| | |
| Robinson | 5 | \| 8039416 \| 384599 \| \| --- \| --- \| | |
| Said | 1 | \| 8043034 \| 389045 \| \| --- \| --- \| | |
| Herbert | 19 | \| 8047696 \| 392041 \| \| --- \| --- \| | |
| Pond | 10 | \| 8051071 \| 392167 \| \| --- \| --- \| | |
| Flying Fish Pt | 5 | \| 8065314 \| 401681 \| \| --- \| --- \| | |
| Satori | 20 | \| 8070295 \| 399537 \| \| --- \| --- \| | |

UTM Grid Coordinates, Zone 55, Datum WGS84
