# Supplementary material for: Feral Pig Populations Are Structured at Fine Spatial Scales in Tropical Queensland, Australia
Source: PLoS One. 2014 Mar 10;9(3):e91657. doi: 10.1371/journal.pone.0091657 (PMC3948871; doi:10.1371/journal.pone.0091657)
Supplement: Table S2 — Genbank accession numbers and pig breed for control region sequences used in Figure 4 (DOCX) [file pone.0091657.s002.docx]

| **Accession No.** | **Breed** | **Reference** |
| --- | --- | --- |
| AB015085 | Japanese Wild Boar | Watonobe *et al.* 1999 |
| AB015087 | Ryuku Wild Boar | Watonobe *et al.* 1999 |
| AB015093 | Yucatan | Watonobe *et al.* 1999 |
| AB015094 | European Wild Boar | Watonobe *et al.* 1999 |
| AB041481 | Moncai | Watonobe *et al.* 2001 |
| AB041484 | Berkshire | Watonobe *et al.* 2001 |
| AF276921 | Westran | Kim *et al.* 2002 |
| AF276922 | Erhualian | Kim *et al.* 2002 |
| AF276923 | Tongcheng | Kim *et al.* 2002 |
| AF276924 | Wanan | Kim *et al.* 2002 |
| AF276925 | Wannanhua | Kim *et al.* 2002 |
| AF276927 | Yanxin | Kim *et al.* 2002 |
| AF276930 | Jinghua | Kim *et al.* 2002 |
| AF276937 | Welsh | Kim *et al.* 2002 |
| AY463064 | Angeln Saddleback | Gongora *et al.* 2004 |
| AY463069 | Landrace | Gongora *et al.* 2004 |
| AY463070 | Large White | Gongora *et al.* 2004 |
| AY463071 | Red Angeln Saddleback | Gongora *et al.* 2004 |
| AY463072 | Mangalitza | Gongora *et al.* 2004 |
| AY463074 | Wessex Saddleback | Gongora *et al.* 2004 |
| AY463075 | Large Black | Gongora *et al.* 2004 |
| AY463090 | Feral (Cooktown QLD) | Gongora *et al.* 2004 |
| AY463091 | Feral (Kowanyama QLD) | Gongora *et al.* 2004 |
| AY463092 | Feral (Julia Creek QLD) | Gongora *et al.* 2004 |
| AY463093 | Feral (Mt Larcom QLD) | Gongora *et al.* 2004 |
| AY463094 | Feral (Vanrook QLD) | Gongora *et al.* 2004 |
| AY463095 | Feral (Rutland QLD) | Gongora *et al.* 2004 |
| AY463096 | Feral (Koolatch QLD) | Gongora *et al.* 2004 |
