# Supplementary material for: Feral Pig Populations Are Structured at Fine Spatial Scales in Tropical Queensland, Australia
Source: PLoS One. 2014 Mar 10;9(3):e91657. doi: 10.1371/journal.pone.0091657 (PMC3948871; doi:10.1371/journal.pone.0091657)
Supplement: Table S3 — Pairwise F ST values. Significant values following Bonferroni correction are highlighted in bold (p<0.0002). (DOCX) [file pone.0091657.s003.docx]

| SITE | MU1 | | | | | | | MU2 | | | | MU3 | | | | | | | | |
| --- | --- | --- | --- | --- | --- | --- | --- | --- | --- | --- | --- | --- | --- | --- | --- | --- | --- | --- | --- | --- |
|  | ITC/  Mac | Condon | Jumbin | Vecchio | Zamora | BSES | Mackay | Agtea | Collins | MissionHts | Krohn | Cowley | CowleyBr | Bay Downs | Ramsay | Smith | Robinson | Herbert | Pond | Flying  Fish |
| Condon | -0.025 |  |  |  |  |  |  |  |  |  |  |  |  |  |  |  |  |  |  |  |
| Jumbin | 0.027 | -0.016 |  |  |  |  |  |  |  |  |  |  |  |  |  |  |  |  |  |  |
| Vecchio | 0.047 | -0.014 | -0.013 |  |  |  |  |  |  |  |  |  |  |  |  |  |  |  |  |  |
| Zamora | 0.032 | -0.008 | 0.026 | 0.016 |  |  |  |  |  |  |  |  |  |  |  |  |  |  |  |  |
| BSES | **0.069** | 0.029 | **0.106** | **0.116** | **0.091** |  |  |  |  |  |  |  |  |  |  |  |  |  |  |  |
| Mackay | **0.071** | 0.046 | 0.090 | **0.095** | **0.159** | **0.205** |  |  |  |  |  |  |  |  |  |  |  |  |  |  |
| Agtea | **0.168** | **0.156** | **0.188** | **0.189** | **0.253** | **0.275** | 0.103 |  |  |  |  |  |  |  |  |  |  |  |  |  |
| Collins | **0.171** | **0.119** | **0.127** | **0.150** | **0.217** | **0.235** | 0.058 | **0.075** |  |  |  |  |  |  |  |  |  |  |  |  |
| MissionHts | **0.141** | **0.055** | **0.085** | **0.094** | **0.175** | **0.203** | 0.069 | **0.109** | **0.033** |  |  |  |  |  |  |  |  |  |  |  |
| Krohn | **0.143** | **0.088** | **0.099** | **0.092** | **0.180** | **0.264** | **0.081** | **0.151** | **0.070** | 0.000 |  |  |  |  |  |  |  |  |  |  |
| Cowley | **0.085** | **0.077** | **0.068** | **0.057** | **0.082** | **0.183** | 0.045 | **0.214** | **0.173** | **0.172** | **0.169** |  |  |  |  |  |  |  |  |  |
| CowleyBr | **0.105** | **0.050** | **0.080** | **0.086** | **0.106** | **0.178** | **0.064** | **0.145** | **0.128** | **0.115** | **0.127** | 0.020 |  |  |  |  |  |  |  |  |
| BayDowns | **0.114** | **0.096** | **0.073** | 0.054 | **0.088** | **0.192** | 0.064 | **0.237** | **0.111** | **0.084** | **0.108** | **0.075** | **0.054** |  |  |  |  |  |  |  |
| Ramsay | **0.082** | **0.118** | 0.037 | -0.031 | **0.062** | **0.201** | 0.024 | **0.270** | **0.164** | **0.101** | **0.090** | 0.019 | **0.055** | **0.098** |  |  |  |  |  |  |
| Smith | **0.099** | 0.069 | 0.063 | 0.074 | 0.073 | 0.179 | 0.084 | **0.244** | **0.140** | 0.114 | **0.113** | 0.066 | 0.080 | 0.014 | 0.054 |  |  |  |  |  |
| Robinson | **0.108** | 0.052 | 0.020 | 0.045 | 0.120 | **0.156** | 0.115 | 0.240 | **0.184** | **0.117** | **0.119** | 0.075 | **0.094** | 0.120 | -0.035 | 0.084 |  |  |  |  |
| Herbert | **0.083** | 0.005 | **0.088** | **0.061** | 0.092 | **0.141** | **0.125** | **0.203** | **0.176** | **0.122** | **0.126** | **0.057** | **0.026** | **0.079** | 0.038 | **0.085** | 0.044 |  |  |  |
| Pond | **0.140** | **0.095** | **0.180** | **0.169** | **0.156** | **0.237** | **0.153** | **0.285** | **0.274** | **0.210** | **0.207** | **0.146** | **0.130** | **0.161** | **0.093** | **0.104** | **0.161** | **0.062** |  |  |
| FlyingFish | **0.098** | 0.041 | 0.068 | 0.085 | **0.106** | 0.127 | 0.113 | 0.150 | **0.157** | **0.120** | **0.149** | 0.090 | 0.056 | 0.082 | 0.114 | 0.096 | 0.034 | 0.053 | **0.188** |  |
| Santori | **0.099** | **0.070** | **0.085** | **0.099** | **0.113** | **0.154** | 0.023 | **0.106** | **0.133** | **0.135** | **0.154** | 0.045 | **0.056** | 0.034 | **0.078** | 0.060 | **0.141** | **0.113** | **0.151** | 0.037 |
